# Chromophobe Renal Cell Carcinoma with Sarcomatoid Differentiation

**DOI:** 10.15586/jkcvhl.v10i3.254

**Published:** 2023-07-08

**Authors:** Benjamin J. Lichtbroun, Brian Shinder, Tina Gowda Sara, Arnav Srivastava, Biren Saraiya, Tina M. Mayer, Ryan Cristelli, Evita Sadimin, Robert E. Weiss, Eric A. Singer

**Affiliations:** 1 Section of Urologic Oncology, Department of Surgery, Rutgers Cancer Institute of New Jersey and Rutgers Robert Wood Johnson Medical School, New Brunswick, NJ, USA;; 2 Division of Medical Oncology, Department of Medicine, Rutgers Cancer Institute of New Jersey and Rutgers Robert Wood Johnson Medical School, New Brunswick, NJ, USA;; 3 Division of Urologic Pathology, Department of Pathology, Rutgers Cancer Institute of New Jersey and Rutgers Robert Wood Johnson Medical School, New Brunswick, NJ, USA

**Keywords:** chromophobe renal cell carcinoma, immune checkpoint inhibitors, kidney cancer, sarcomatoid

## Abstract

Chromophobe renal cell carcinoma (chRCC) is one of the less common types of kidney cancer and generally portends a more favorable prognosis. RCC with sarcomatoid differentiation has a more aggressive clinical course with poor outcomes. Four cases of chRCC with varying degrees of sarcomatoid differentiation were retrospectively reviewed at our institution, and clinicopathologic data as well as clinical courses were reported. Patients with higher degrees of sarcomatoid differentiation and larger tumors at presentation generally had and worse overall survival. chRCC with sarcomatoid differentiation portends a poor prognosis with limited data on systemic treatment options for metastatic disease.

## Introduction

Renal cell carcinoma (RCC) remains one of the most commonly diagnosed malignancies in the United States (1). Many histologic subtypes of RCC have been described, with the clear cell RCC (ccRCC) variation accounting for nearly 75% of cases (2). Chromophobe renal cell carcinoma (chRCC) is less common, diagnosed in only 5–10% of renal tumors, and typically confers a more indolent course (3, 4). The presence of sarcomatoid differentiation in RCC, which is seen in approximately 5–10% of cases, is typically associated with a highly aggressive behavior, rapid recurrence, a predilection for metastasis, and an extremely poor prognosis (5). Though sarcomatoid features are most commonly found in ccRCC, small subsets of chRCC tumors harbor these pathologic changes as well.

Available evidence seems to suggest that sarcomatoid differentiation in chRCC confers a poor prognosis, though given the scarcity of this subtype, data is limited (6–8). Thus, optimal treatment pathways are unknown. This report highlights four cases of chRCC with sarcomatoid differentiation at a single institution to add to the body of literature describing this clinical entity.

## Methods

Four cases of chRCC with varying degrees of sarcomatoid differentiation were retrospectively reviewed at our institution. Clinicopathologic data and clinical courses are reported.

## Results

### Case 1

A 47-year-old male developed left-sided flank pain and was found to be anemic with a large left-sided palpable mass in 2010. Computed tomography (CT) revealed an 18 cm leftsided lower pole renal mass with possible colonic involvement and no evidence of metastatic disease. He underwent an open left radical nephrectomy, lymphadenectomy, and bowel resection. Surgical pathology was chRCC with 20% sarcomatoid component. The tumor invaded into the perinephric fat, Gerotas fascia, and into the subserosa of the large bowel. The bowel however was uninvolved by tumor. One lymph node was removed and was negative for tumor. Final pathology was T4N0M0. He had an uneventful postoperative course. Due to high risk of progression, he was treated with sunitinib at an outside institution. At our institution, he was started on erlotinib with a PI3K inhibitor (pictilisib) as part of a phase I clinical trial (9). Two months after starting the therapy and 11 months post operation, the patient was noted to have multiple large retroperitoneal masses and new lung nodules, indicating progression of disease and therefore was switched to everolimus. Fine needle aspiration showed poorly differentiated carcinoma. The disease continued to progress, and the patient ultimately died 15 months after his initial surgery at an outside hospital.

### Case 2

A 51-year-old female initially presented with right flank and chest pain in 2016. Workup for which revealed a 29 cm right renal mass believed to be eroding into the liver. The mass was biopsied percutaneously showing sarcomatoid tumor. It was unclear at that time if it was RCC or urothelial carcinoma. She was also found to have a right pleural effusion requiring chest tube placement, with cytology from the pleural fluid negative for malignancy. Further workup included a bone scan and MRI brain, which did not reveal any evidence of metastatic disease. She underwent an open right radical nephrectomy with en bloc right hepatectomy, resection of right hemidiaphragm, retroperitoneal lymphadenectomy, and cholecystectomy. Pathology was chRCC with 95% sarcomatoid element and direct extension into the adrenal gland and liver, with 3 out of 11 lymph nodes positive for a final pathologic stage of T4N1M0. There was a positive posterior and vascular margin. Her postoperative course was complicated by a pulmonary embolism, biliary leak, vancomycinresistant enterococci (VRE) bacteremia, and delirium. She was discharged to rehab on postoperative day 24 and returned 2 days later due to a desaturation event at the rehab accompanied by fevers. Repeated CT showed progression of disease with peritoneal implants along the ascending colon and cecum, a mass on the right psoas muscle, and multiple pleural-based masses along the right costophrenic angle. The patient developed urosepsis and subsequent septic shock and died 60 days after the index procedure.

### Case 3

A 63-year-old male initially presented with 3 weeks of severe abdominal pain in 2017. Workup revealed a 26 cm renal mass involving the lower pole of the right kidney with no evidence of metastatic disease. The mass was biopsied percutaneously, which showed only chRCC. During the same hospital stay, he underwent an open right radical nephrectomy that necessitated a right hemicolectomy with a small bowel resection with primary anastomosis. Pathology was chRCC with 65% sarcomatoid element, with 0 out of 2 lymph nodes positive for a final pathologic stage of T4N0M0. There were negative margins. Four months after the index surgery, he developed significant abdominal distension. CT showed a new necrotic 17 cm peritoneal mass as well as significant ascites and a small bowel obstruction. He was started on total parenteral nutrition and was given a venting PEG tube. The patient received one cycle of nivolumab prior to being admitted. Five weeks after starting nivolumab and 161 days after his initial surgery, the patient died of septic shock of unclear etiology.

### Case 4

A 51-year-old female initially was noted to have an incidental left renal mass on workup for appendicitis in 2015. At that time, it was biopsied at an outside hospital and was interpreted as an oncocytoma. In 2018, her primary care physician noted a mass in the left upper quadrant during a routine visit and sent her for a CT, which was notable for a 15 cm lower pole renal mass with bulky left retroperitoneal adenopathy. Further workup included a brain MRI, CT chest, and nuclear medicine bone scan, which was notable for only a small pericardial effusion and also an area of questionable uptake in the left lower sternum (no evidence of bony metastasis on CT imaging). A repeat percutaneous biopsy was performed, which showed only chRCC. She ultimately underwent an open left radical nephrectomy, retroperitoneal lymphadenectomy, and umbilical hernia repair. Pathology was chRCC with focal sarcomatoid features. A large 19 cm lymph node packet in the preand para-aortic regions was positive for chRCC for a final stage of T3aN1. Surgical margins were negative. She had an uneventful postoperative course. Follow-up imaging 14 months post operation revealed new retroperitoneal lymphadenopathy with a 1.4 cm lesion posterior to the left psoas as well as new adenopathy in the neck and mediastinum region. Biopsy confirmed recurrence of the disease. She was started on pembrolizumab and axitinib shortly after the diagnosis of disease recurrence and was maintained on it for 2 years—200 mg pembrolizumab every 3 weeks and 5 mg axitinib daily. Restaging scan 2 years after starting systemic therapy showed worsening abdominopelvic and thoracic lymphadenopathy, prompting a treatment change to cabozantinib at her last visit, that is, 40 mg daily. At last follow-up, the patient is 44 months from her index surgery.

### Histology and immunochemistry

**Figure 1:**
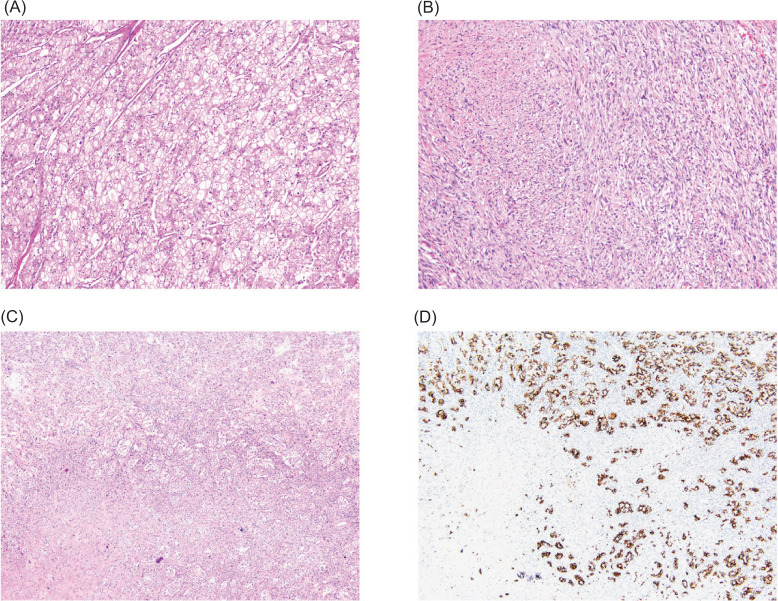
(A) Area of the tumor showing more typical chromophobe renal cell carcinoma morphology, low magnification; (B) Area of the tumor showing the sarcomatoid morphology, low magnification; (C) Transition area with (D) CK7 immunostain highlighting the chromophobe component while negative in the sarcomatoid component.

In cases 2 and 3, the tumors showed areas with classic chRCC morphology (Figure 1A), composed of oncocytic cells with abundant clear to granular cytoplasm, perinuclear clearing, and irregular nuclear contours. The sarcomatoid areas showed spindle cells with fascicular and storiform growth patterns (Figure 1B). At the area of transition from the classical morphology to the sarcomatoid morphology (Figure 1C) in one of our cases, the sarcomatoid component showed loss of CK7 immunostaining, which is diffusely positive in the adjacent chromophobe component (Figure 1D). Both tumors had extensive necrosis and extended beyond the kidney. In Case 2, the tumor invaded through the kidney, through the adjacent adrenal gland, and into the liver. Multiple lymph nodes were positive for metastasis. While in Case 3, the tumor invaded through the kidney into the perinephric tissue, adjacent small bowel wall, and into the mesentery. There was no lymph node involvement.

## Discussion

While ccRCC is the most common histologic subtype of RCC, chRCC comprises roughly 5–10% of the cases and generally portends a better prognosis (3, 4). Localized chRCC is generally treated with surgery and at 5 years has a recurrence-free survival (RFS) of 89.3% and a cancer-specific survival (CSS) rate of 93% (10). chRCC with sarcomatoid features generally portends a poor prognosis when compared to the relatively indolent nature of chRCC (3, 4, 6, 8, 11). While chRCC is one of the less common subtypes of RCC, the combination of chRCC with sarcomatoid differentiation presents a uniquely rare clinical entity. Because of the relative rarity of metastatic chRCC, prospective trials are generally not available, and patients with this clinical entity are often grouped together as “non-ccRCC.” While there have been many advances in the treatment of metastatic ccRCC, therapeutic advances in non-ccRCC have been somewhat limited due to low incidence and limited clinical trial success (12). The National Comprehensive Cancer Network (NCCN) recommends enrollment in a clinical trial for patients with metastatic non-ccRCC with other preferred regimens including tyrosine kinase inhibitors such as cabozantinib and sunitinib (13).

In this case series, we discuss four patients with chRCC with sarcomatoid features who presented in their fourth through sixth decades of life (Tables 1 and 2). In our cohort, patients who had a larger percentage of sarcomatoid differentiation generally had a shorter overall survival (OS), while patients who had a small percentage of sarcomatoid component generally lived longer. One patient had only focal sarcomatoid component and is still alive 44 months after surgery. On the other hand, a patient with 95% sarcomatoid component died 60 days following surgery, and she had a complicated postoperative course with early recurrence. In addition, our patients who had a larger sarcomatoid component also had a larger tumor size at presentation. These findings are similar to the previous study reports on chRCC with sarcomatoid differentiation (6–8).

**Table 1:** Patient, clinical, and pathologic characteristics of four patients with chRCC with sarcomatoid features.

	Patient 1	Patient 2	Patient 3	Patient 4
Age	47	51	63	51
Gender	Male	Female	Male	Female
Clinical stage	T2b	T4	T2b	T2b
Size of primary tumor	18 cm	29 cm	26 cm	15 cm
Surgical procedure	Open left radical nephrectomy, lymphadenectomy, bowel resection	Open right radical nephrectomy, en bloc right hepatectomy, resection of right hemidiaphragm, retroperitoneal lymphadenectomy, cholecystectomy	Open right radical nephrectomy, right hemicolectomy, small bowel resection, and primary anastomosis	Open left radical nephrectomy, retroperitoneal lymphadenectomy, umbilical hernia repair
Surgical pathology	Chromophobe RCC	Chromophobe RCC	Chromophobe RCC	Chromophobe RCC
% Sarcomatoid	20%	95%	65%	focal
Surgical stage	T4N0	T4N1	T4N0	T3aN1
Additional systemic therapy	Suntinib at outside institution, erlotinib and PI3K inhibitor (pictisilib), everolimus	None	Nivolumab	Pembrolizumab and axitinib, cabozantinib
Location of metastasis	Multiple large retroperitoneal masses, new lung nodules	Peritoneal implants along the ascending colon/cecum, mass on right psoas muscle, multiple pleural-based masses along the right costophrenic angle	Necrotic 17 cm peritoneal mass, significant ascites, small bowel obstruction	Retroperitoneal lymphadenopathy,1.4 cm lesion posterior to the left psoas, adenopathy in the neck and mediastinum
Time from surgery to first recurrence	11 months	29 days	4 months	14 months
Time from surgery to death	15 months	60 days	161 days	N/A

RCC, renal cell carcinoma.

**Table 2:** Summary of existing literature on patients with chRCC with sarcomatoid features within the last 10 years.

Study	N	Primary endpoint	Outcome	Notes
Ged et al.	29	RFS	RFS was shorter in patients with sarcomatoid features than without – 2.7 months (95% CI, 0.7–6.9) vs 48.8 months (95% CI, 30.8–80.7, P < 0.001)	Retrospective review of 109 patients with metastatic chRCC. 29 patients with a sarcomatoid component were compared to those without
Pieretti et al.	10	CSS, RFS	10-year RFS was 91.4% in high risk group and 34.4% in low risk group (P < 0.001). 10-year CSS was 96.4% in the low-risk group and 54.3% in the high risk group (P < 0.001). Sarcomatoid features was independently associated with RFS (HR 5.5, 95% CI 1.5–20.2, P = 0.01)	Retrospective review of 300 patients with sporadic, unilateral, nonmetastatic chRCC. Patients with sarcomatoid features or nodal disease were considered high risk
Casuscelli et al.	6	RFS, OS	In univariate analysis with patients with sarcomatoid differentiation, OS (HR 38.686, 95% CI 14.721–101.662, P < 0.001) and RFS (HR 40.747, 13.363–124.245, P < 0.001) were both significantly shorter	A prospectively maintained database was queried to compare 3312 patients with ccRCC to 496 patients with chRCC who were surgically treated and clinicopathologic characteristics were compared
Volpe et al.	5	RFS, CSS	On univariable analysis, sarcomatoid differentiation was associated with decreased CSS (HR 25.9, 95% CI 8.1–83.1, P < 0.001) and decreased RFS (HR 18.2, 95% CI 6–55, P < 0.001)	291 patients with chRCC were identified in a retrospective database of patients surgically treated for RCC from 1995 to 2007, and cancerrelated outcomes and prognostic factors for chRCC were assessed
Cheville et al.	13	CSS	On univariate analysis, sarcomatoid differentiation was associated with decreased CSS (HR 45.88, 95% CI 15.55–135.38, P < 0.001)	Pathologic features of 185 patients with chRCC who were surgically treated between 1970 and 2006 were reviewed
Lauer et al.	14	OS	10 out of 14 patients died of the disease. 9 died within the first 6 months since surgery; mean survival of 10 weeks.	Surgical pathology from 14 patients with chRCC with sarcomatoid features were retrospectively reviewed and clinicopathologic features assessed
Przbycin et al.	4	5-year cumulative incidence of events – local recurrence or metastasis	1 out of the 4 patients had a metastasis and zero had a local recurrence (HR 33.3, 95% CI 0.9–77.4, P = 0.02)	A prospectively maintain database was queried and 203 patients with chRCC who were surgically treated between 1988 and 2006 were identified and clinicopathologic characteristics were assessed

ccRCC, clear cell renal cell carcinoma; chRCC, chromophobe renal cell carcinoma; CSS, cancer-specific survival; OS, overall survival; RFS, recurrence-free survival.

In a recent study by Casuscelli et al., they reviewed the clinicopathological parameters of all patients diagnosed with chRCC between 1990 and 2016 and compared them to patients with ccRCC. There were a total of 496 patients with chRCC and 3312 patients with ccRCC included. Their findings revealed that patients with larger tumors and those with sarcomatoid differentiation were more likely to have metastatic development, decreased RFS, and decreased OS (11). A multicenter dataset evaluated prognostic factors for RFS and CSS in patients with chRCC. Out of 5463 patients surgically treated for RCC, 91 had chRCC. They found that in addition to male gender and stage of the disease, sarcomatoid differentiation was an independent predictor of worse RFS and CSS (10).

Previous studies also found that in patients with RCC, the percentage of sarcomatoid component was associated with poorer outcomes (14). A recent study by Ged et al. retrospectively assessed patients with metastatic chRCC and compared outcomes between patients with and without sarcomatoid differentiation. They found that after nephrectomy, the sarcomatoid group had quicker time to treatment failure and decreased OS (38 months vs 7.5 months) (6). A study by Pieretti et al. looked at the long-term outcomes in patients with chRCC. They placed patients with sarcomatoid RCC or lymph node–positive disease into the high-risk category and compared them to patients without these findings. They found that only 10 patients out of 300 had sarcomatoid differentiation. They found that the high-risk group had higher risk of recurrence, that is, 50% versus 4.9%; lower 10-year RFS, that is, 91.4% versus 34.4%; and lower 10-year CSS, that is, 96.4% versus 54.3% (15).

As various chemotherapeutic regimens have shown efficacy in other types of sarcomas, multiple studies have attempted to extrapolate these findings to sarcomatoid RCC. The combination of gemcitabine and doxorubicin has shown some activity in patients with RCC with sarcomatoid features (16, 17). Sunitinib in combination with gemcitabine was also investigated in patients with sarcomatoid or poorrisk metastatic RCC, and the authors found that the combination of both medications was better than either one alone (18). The combination of bevacizumab with capecitabine and gemcitabine is also being investigated—although there are low response rates, it has been well tolerated (18, 19).

While sarcomatoid differentiation generally portends a worse prognosis in RCC, it does have a unique immunologic landscape with frequent expression of program death ligand-1 (PD-L1) (20). Targeting this pathway has shown clinical benefit in the setting of metastatic ccRCC with sarcomatoid features, making this an attractive target for future clinical trials for metastatic chRCC (20, 21). It is also worth noting that the one patient in our cohort who received a combination of pembrolizumab and axitinib is still alive 44 months after surgery. While she of course only had focal sarcomatoid features, it highlights the recent advances in systemic therapy in treating these patients. With the treatment landscape for metastatic and advanced RCC changing, including immune checkpoint inhibitors (ICI) as a staple of treatment, there has been much discussion surrounding the use of these treatment modalities for patients with sarcomatoid differentiation. A recent meta-analysis and systematic review evaluated the six published Phase III randomized controlled trials evaluating ICI-based combination therapy for metastatic RCC. Five out of these six studies presented data on the subset of patients with ccRCC with sarcomatoid features, a total of 568 patients. In all five studies, there was a significantly improved progression-free survival (PFS) and improved objective response rate with combination ICI therapy compared to sunitinib. The combination of nivolumab and ipilimumab achieved the highest complete response rate, and the combination of nivolumab and cabozantinib had the highest likelihood of improvement in PFS and OS (22). Unfortunately, it remains to be seen whether such treatment advances are applicable to patients with chRCC and sarcomatoid differentiation given the relative rarity of this combination. Sarcomatoid and rhabdoid RCC tumors have also been found to have unique molecular markers, which may account for their aggressive nature. In addition to their unique genomic and transcriptomic features, these tumors also exhibit an immune-inflamed phenotype, which may account for the responsiveness to ICIs (23). With the positive results in these recent studies, it begs the question whether a deferred surgery should be considered in patients who are excellent responders to one of these combination therapies or if these medications should be considered largely palliative.

The exact mechanism of sarcomatoid dedifferentiation is unclear. One theory, called the epithelial-mesenchymal transition (EMT), posits that tumors with a sarcomatoid component have two separate cell lines within the tumor—an epithelial component and a mesenchymal component (24). Evidence suggests that the mesenchymal component, which gives rise to the sarcomatoid features, starts from the same cell line as the epithelial component. This process is seen in normal development in order to create different types of specialized tissue; however, it can also be seen with tumor development (24).

This data also highlights the limitations of percutaneous renal mass biopsy, as one of our patients was noted to have chRCC on biopsy and it was not until after surgery that he or she was found to have a significant percentage of sarcomatoid component. A second patient had her initial biopsy at an outside hospital, which was thought to be an oncocytoma and 3 years later had a repeat biopsy that revealed only chRCC. Given that percutaneous renal mass biopsies sample a small area, future research will need to investigate better imaging modalities in order to detect sarcomatoid features (25). Physicians should also have a high level of suspicion for sarcomatoid features when the renal mass biopsy shows a more indolent pathology, but the tumor biology appears aggressive in nature with a large tumor burden, lymphadenopathy, or metastatic disease.

Much of the data relating to RCC with sarcomatoid features is in patients with clear cell pathology. chRCC generally portends a more favorable prognosis with different tumor biology, making comparisons between the two histologies challenging. While ICIs for ccRCC with sarcomatoid features has shown promise in the metastatic setting, there is limited data on this in the adjuvant setting, making this an opportunity for further research (26). As we begin to understand the tumor molecular markers of sarcomatoid RCC, it is important to note the differences between clear cell and non-clear cell pathology.

There are multiple ongoing trials evaluating whether ICIs may be beneficial for patients was RCC with sarcomatoid features. This cohort also highlights the need for improved detection of sarcomatoid features as percutaneous renal mass biopsy and current imaging modalities often are unable to detect this unfavorable pathology. Future trials are needed to specifically evaluate patients with non-clear cell RCC and those with sarcomatoid differentiation. Further, clinicopathologic and translational aspects of chRCC need to be studied to aid in future prognosis and management of these patients (27–30). The affected patients may also benefit from adjuvant therapy given the high risk of disease recurrence after surgery.

## Conclusion

chRCC is a less common form of kidney cancer that is often very indolent and can be treated with surgical resection. An even less common clinical scenario is when patients with chRCC have components of sarcomatoid differentiation, which has a much more aggressive course. Here, we presented four patients in their fourth to sixth decades of life, who had chRCC with varying degrees of sarcomatoid component. In our patients, it was observed that those who had a larger degree of sarcomatoid component and larger tumor size at presentation had a worse outcome, which is in line with recent reports. Future clinical trials are required in order to determine optimal systemic therapy regimens for these patients.

## References

[1] Miller KD, Nogueira L, Devasia T, Mariotto AB, Yabroff KR, Jemal A, et al. Cancer treatment and survivorship statistics, 2022. CA Cancer J Clin. 2022;72(5):409–36. 10.3322/caac.21731 35736631

[2] Shuch B, Amin A, Armstrong AJ, Eble JN, Ficarra V, LopezBeltran A, et al. Understanding pathologic variants of renal cell carcinoma: Distilling therapeutic opportunities from biologic complexity. Eur Urol. 2015;67(1):85–97. 10.1016/j.eururo.2014.04.029 24857407

[3] Cheville JC, Lohse CM, Zincke H, Weaver AL, Blute ML. Comparisons of outcome and prognostic features among histologic subtypes of renal cell carcinoma. Am J Surg Pathol. 2003;27(5):612–24. 10.1097/00000478-200305000-00005 12717246

[4] Amin MB, Paner GP, Alvarado-Cabrero I, Young AN, Stricker HJ, Lyles RH, et al. Chromophobe renal cell carcinoma: Histomorphologic characteristics and evaluation of conventional pathologic prognostic parameters in 145 cases. Am J Surg Pathol. 2008;32(12):1822–34. 10.1097/PAS.0b013e3181831e68 18813125

[5] Mouallem NE, Smith SC, Paul AK. Sarcomatoid renal cell carcinoma: Biology and treatment advances. Urol Oncol. 2018;36(6):265–71. 10.1016/j.urolonc.2017.12.012 29306556

[6] Ged Y, Chen YB, Knezevic A, Casuscelli J, Redzematovic A, DiNatale RG, et al. Metastatic chromophobe renal cell carcinoma: Presence or absence of sarcomatoid differentiation determines clinical course and treatment outcomes. Clin Genitourin Cancer. 2019;17(3):e678–e88. 10.1016/j.clgc.2019.03.018 31036466 PMC6752712

[7] Cheville JC, Lohse CM, Sukov WR, Thompson RH, Leibovich BC. Chromophobe renal cell carcinoma: The impact of tumor grade on outcome. Am J Surg Pathol. 2012;36(6):851–6. 10.1097/PAS.0b013e3182496895 22367296

[8] Lauer SR, Zhou M, Master VA, Osunkoya AO. Chromophobe renal cell carcinoma with sarcomatoid differentiation: A clinicopathologic study of 14 cases. Anal Quant Cytopathol Histpathol. 2013;35(2):77–84. 23700716

[9] Leong S, Moss RA, Bowles DW, Ware JA, Zhou J, Spoerke JM, et al. A phase I dose-escalation study of the safety and pharmacokinetics of pictilisib in combination with erlotinib in patients with advanced solid tumors. Oncologist. 2017;22(12):1491-9. 10.1634/theoncologist.2017-0090 28798270 PMC5728021

[10] Volpe A, Novara G, Antonelli A, Bertini R, Billia M, Carmignani G, et al. Chromophobe renal cell carcinoma (RCC): Oncological outcomes and prognostic factors in a large multicentre series. BJU Int. 2012;110(1):76–83. 10.1111/j.1464-410X.2011.10690.x 22044519

[11] Casuscelli J, Becerra MF, Seier K, Manley BJ, Benfante N, Redzematovic A, et al. Chromophobe renal cell carcinoma: Results from a large single-institution series. Clin Genitourin Cancer. 2019;17(5):373–9 e4. 10.1016/j.clgc.2019.06.011 31326335 PMC6790280

[12] Patel HV, Srivastava A, Srinivasan R, Singer EA. A challenging frontier—The genomics and therapeutics of nonclear cell renal cell carcinoma. Curr Opin Oncol. 2021;33(3):212–20. 10.1097/CCO.0000000000000721 33818540 PMC8244822

[13] Motzer RJ, Jonasch E, Agarwal N, Alva A, Baine M, Beckermann K, et al. Kidney cancer, version 3.2022, NCCN clinical practice guidelines in oncology. J Natl Compr Canc Netw. 2022;20(1):71–90. 10.6004/jnccn.2022.0001 34991070 PMC10191161

[14] de Peralta-Venturina M, Moch H, Amin M, Tamboli P, Hailemariam S, Mihatsch M, et al. Sarcomatoid differentiation in renal cell carcinoma: A study of 101 cases. Am J Surg Pathol. 2001;25(3):275–84. 10.1097/00000478-200103000-00001 11224597

[15] Pieretti AC, Westerman ME, Childs A, Millward N, Shapiro DD, Sircar K, et al. Sarcomatoid features and lymph node-positive disease in chromophobe renal cell carcinoma. Urol Oncol. 2021;39(11):790 e17–e23. 10.1016/j.urolonc.2021.06.016 34301458

[16] Haas NB, Lin X, Manola J, Pins M, Liu G, McDermott D, et al. A phase II trial of doxorubicin and gemcitabine in renal cell carcinoma with sarcomatoid features: ECOG 8802. Med Oncol. 2012;29(2):761–7. 10.1007/s12032-011-9829-8 21298497 PMC3566570

[17] Roubaud G, Gross-Goupil M, Wallerand H, de Clermont H, Dilhuydy MS, Ravaud A. Combination of gemcitabine and doxorubicin in rapidly progressive metastatic renal cell carcinoma and/or sarcomatoid renal cell carcinoma. Oncology. 2011;80(3-4):214–8. 10.1159/000329078 21720184

[18] Motzer RJ, Jonasch E, Boyle S, Carlo MI, Manley B, Agarwal N, et al. NCCN guidelines insights: Kidney cancer, Version 1.2021. J Natl Compr Canc Netw. 2020;18(9):1160–70. 32886895 10.6004/jnccn.2020.0043 PMC10191771

[19] Maiti A, Nemati-Shafaee M, Msaouel P, Pagliaro LC, Jonasch E, Tannir NM, et al. Phase 2 trial of capecitabine, gemcitabine, and bevacizumab in sarcomatoid renal-cell carcinoma. Clin Genitourin Cancer. 2017;S1558-7673(17):30238. 10.1016/j.clgc.2017.07.028 PMC5809227 28870517

[20] Moch H, Ohashi R. Chromophobe renal cell carcinoma: Current and controversial issues. Pathology. 2021;53(1):101–8. 10.1016/j.pathol.2020.09.015 33183792

[21] Chahoud J, Msaouel P, Ross JA, McCormick BZ, Bathala TK, Gao J, et al. Outcomes of patients with metastatic renal cell carcinoma with sarcomatoid dedifferentiation to immune checkpoint inhibitors. Urol Oncol. 2021;39(2):134 e9–e16. 10.1016/j.urolonc.2020.10.019 33187886

[22] Quhal F, Mori K, Fajkovic H, Remzi M, Shariat SF, Schmidinger M. Immunotherapy-based combinations in the first-line treatment of metastatic renal cell carcinoma with sarcomatoid features: A systematic review and network meta-_analysis. Curr Opin Urol. 2022;32(1):61–8. 10.1097/MOU.0000000000000940 34720102

[23] Bakouny Z, Braun DA, Shukla SA, Pan W, Gao X, Hou Y, et al. Integrative molecular characterization of sarcomatoid and rhabdoid renal cell carcinoma. Nat Commun. 2021;12(1):808. 10.1038/s41467-021-21068-9 33547292 PMC7865061

[24] Blum KA, Gupta S, Tickoo SK, Chan TA, Russo P, Motzer RJ, et al. Sarcomatoid renal cell carcinoma: biology, natural history and management. Nat Rev Urol. 2020;17(12):659–78. 10.1038/s41585-020-00382-9 33051619 PMC7551522

[25] Wu Y, Kwon YS, Labib M, Foran DJ, Singer EA. Magnetic resonance imaging as a biomarker for renal cell carcinoma. Dis Markers. 2015;2015:648495. 10.1155/2015/648495 26609190 PMC4644550

[26] Patel HD, Puligandla M, Shuch BM, Leibovich BC, Kapoor A, Master VA, et al. The future of perioperative therapy in advanced renal cell carcinoma: How can we PROSPER? Future Oncol. 2019;15(15):1683–95. 10.2217/fon-2018-0951 30968729 PMC6595543

[27] Alaghehbandan R, Przybycin CG, Verkarre V, Mehra R. Chromophobe renal cell carcinoma: Novel molecular insights and clinicopathologic updates. Asian J Urol. 2022;9(1):1–11. 10.1016/j.ajur.2021.11.010 35198391 PMC8841285

[28] Chen CV, Croom NA, Simko JP, Stohr BA, Chan E. Differential immunohistochemical and molecular profiling of conventional and aggressive components of chromophobe renal cell carcinoma: Pitfalls for diagnosis. Hum Pathol. 2022;119:85–93. 10.1016/j.humpath.2021.11.003 34800526

[29] Przybycin CG, Cronin AM, Darvishian F, Gopalan A, Al-Ahmadie HA, Fine SW, et al. Chromophobe renal cell carcinoma: A clinicopathologic study of 203 tumors in 200 patients with primary resection at a single institution. Am J Surg Pathol. 2011;35(7):962–70. 10.1097/PAS.0b013e31821a455d 21602658

[30] Pichler R, Comperat E, Klatte T, Pichler M, Loidl W, Lusuardi L, et al. Renal cell carcinoma with sarcomatoid features: Finally, new therapeutic hope? Cancers (Basel). 2019;11(3):422. 10.3390/cancers11030422 30934624 PMC6468799

